# Dietary fermented rapeseed or/and soybean meal additives on performance and intestinal health of piglets

**DOI:** 10.1038/s41598-021-96117-w

**Published:** 2021-08-20

**Authors:** Anna Czech, Eugeniusz Ryszard Grela, Martyna Kiesz

**Affiliations:** 1 Department of Biochemistry and Toxicology, Faculty of Animal Sciences and Bioeconomy, University of Life Sciences in Lublin, Akademicka 13, 20-950 Lublin, Poland; 2 Institute of Animal Nutrition and Bromatology, Faculty of Animal Sciences and Bioeconomy, University of Life Sciences in Lublin, Akademicka 13, 20-950 Lublin, Poland

**Keywords:** Biochemistry, Biotechnology, Microbiology

## Abstract

The aim of the study was to assess the effect of fermented dried soybean (FSBM) and/or fermented rapeseed meal (FRSM) in diets for weaned piglets on production results, nutrient digestibility, gastrointestinal tract histology, and the composition of the gut microbiota. Piglets in the control group received standard diets with soybean meal. Animals in all experimental groups received diets in which a portion of the soybean meal was replaced: in group FR—8% FRSM; in group FR/FS—6% FRSM and 2% FSBM; in group FS/FR—2% FRSM and 6% FSBM and in group FS—8% FSBM. The use of 8% FRSM or 6% FRSM and 2% FSBM in the piglet diets had a positive effect on average daily gains. Piglets from the FR and FR/FS groups had the highest feed conversion rate. Group FS/FR and FS piglets had significantly lower mortality and lower incidence of diarrhoea. Piglets fed a diet with the fermented components, in particular with 8% FRSM or 6% FRSM and 2% FSBM, exhibited a positive effect on the microbiological composition and histology of intestines, which resulted in improved nutrient digestibility coefficients (ATTD and AID).

## Introduction

Due to the ban on the use of feed derived from mammals and poultry in livestock feeding, the most common protein component of feed is soybean meal (SBM). It has high protein content, a balanced amino acid profile, except for sulphur-containing amino acids^1^, and low content of anti-nutritional substances^2^. Widespread use of this feed component, especially in the diet of young animals, is limited by the presence of β-conglycinin and non-starch polysaccharides, such as raffinose and stachyose^3,4^, which are not digested in the ileum of monogastric animals due to the lack of specific enzymes. In addition, soybean seeds are mainly obtained from genetically modified (GMO) crops, accounting for as much as 64% of all protein feed used in Europe^1^.

There is currently a search for alternative protein components to replace GMO soybean meal in diets for pigs and poultry. Livestock farmers are showing interest in legume seeds (e.g. lupin, broad beans, and peas) as well as products of the oil industry, i.e. rapeseed meal (RSM) and rapeseed cake. In comparison with soybean, rapeseed protein contains more sulphur-containing amino acids (methionine and cysteine), the deficiency of which limits the utilization of plant feed in diets for pigs. On the other hand, rapeseed meal has lower content of protein, including lysine, higher content of dietary fibre, and thus a lower caloric value. Moreover, it contains anti-nutritional compounds such as glucosinolates, tannins, phytates, and others^5^. Various methods are used to improve the nutritional value of both rapeseed meal and soybean meal, such as toasting, extrusion, or sieve separation (reducing the level of fibre). One of the more interesting methods is fermentation, during which microbes break down anti-nutritional substances^2^, induce synthesis of various bioactive compounds, primarily antioxidants^6,7^, reduce the content of raw fibre, and modify the protein amino acid profile, which leads to better production parameters in pig farming^8^. Thus far, feed fermentation has been used mainly in liquid feeding systems^8,9^. However, as many farms use complete dry or pelleted feed, it would be interesting to analyse the effectiveness of using fermented dried soybean and/or rapeseed meal in the diet for piglets and weaners. We hypothesize that the use of fermented dried rapeseed meal or/and soybean meal, with increased nutritional value and reduced content of anti-nutritional substances, may improve the body condition and growth performance of weaned piglets and modify their gut microbiota.

## Results

### Chemical composition of fermented components and diets for piglets

The content of crude protein, lysine, tryptophan, threonine, copper, and iron and metabolizable energy was higher in the fermented soybean meal (FSBM) than in the fermented rapeseed meal (FRSM). The reverse was noted for crude ash, crude fibre, total phosphorus, phytate phosphorus, calcium, sodium and zinc. FSBM also had lower content of glucosinolates and tannins^10^ (Table 1).

**Table 1:** Content of analysed nutrients and bioactive substances in 1 kg of FRSM (n = 4) and FSBM (n = 4) and piglet diets (n = 3)^10^.

Item	FRSM	FSBM	Prestarter (29–42 days)					Starter (43–77 days)				
			C	FR	FR/FS	FS/FR	FS	C	FR	FR/FS	FS/FR	FS
Dry matter (g)	882.7	901.1	891.4	889.5	890.2	889.9	889.8	890.2	888.9	889.4	889.5	889.1
Crude ash (g)	78.9	66.1	50.68	50.67	50.66	50.69	50.72	50.42	50.51	50.41	50.56	50.57
Crude protein (g)	291.8	499.7	187.84	186.95	187.45	188.03	188.11	181.01	181.11	181.09	181.07	181.09
Ether extract (g)	31.7	35.6	50.31	50.32	50.34	50.35	40.33	50.13	50.18	50.14	50.15	40.13
Crude fibre (g)	91.5	17.9	40.07	40.05	40.05	40.03	40.02	40.41	40.28	40.22	39.98	39.95
Metabolizable energy^a^ (MJ)	12.27	15.64	13.32	13.32	13.31	13.32	13.32	13.21	13.21	13.21	13.21	13.21
Total phosphorus (g)	9.09	7.12	6.81	6.82	6.82	6.81	6.81	6.78	6.75	6.78	6.76	6.77
Phytin phosphorus (g)	5.73	4.61	3.92	2.44	2.73	2.82	2.74	3.93	2.46	2.76	2.87	2.79
Calcium (g)	8.05	2.54	7.41	7.41	7.42	7.43	7.43	7.38	7.37	7.38	7.39	7.38
Sodium (g)	2.26	0.21	2.12	2.12	2.12	2.12	2.13	2.11	2.11	2.12	2.11	2.11
Zinc (mg)	66.98	47.37	148.7	149.4	149.3	149.5	149.6	148.2	149.3	148.8	148.7	149.2
Copper (mg)	6.67	14.98	161.3	161.5	161.7	161.6	161.4	161.4	161.6	161.9	161.8	161.5
Iron (mg)	149.2	191.4	159.3	161.8	161.7	161.6	161.5	159.4	162.1	161.9	161.8	161.7
Total lysine (g)	13.55	24.22	13.6	13.7	13.8	13.7	13.8	12.5	12.8	12.7	12.6	12.9
Methionine (g)	5.48	6.43	3.89	3.89	3.88	3.89	3.91	3.82	3.81	3.82	3.81	3.82
Threonine (g)	12.24	16.79	8.64	8.65	8,64	8.63	8.64	8.51	8.43	8,43	8.45	8.44
Tryptophan (g)	2.76	5.70	2.54	2.52	2.51	2.53	2.53	2.52	2.49	2.49	2.51	2.51
Glucosinolates (µmol/g)	6.37	0.92	0.081	0.330	0.174	0.103	0.062	0.081	0.331	0.175	0.104	0.063
Tannin (g/kg)	4.76	1.22	2.41	3.16	2.81	2.58	2.39	2.44	3.17	2.82	2.59	2.40
Lactic acid (g/kg)	50.42	44.22	1.47	5.43	5.32	5.08	4.95	1.43	5.32	5.29	5.06	4.89

*FSBM* fermented dried soybean meal, *FRSM* fermented dried rapeseed meal.

Metabolizable energy^a^ was calculated according to the equation proposed by Kirchgessner and Roth^11^.

^a^Feeding groups: C—control; FR- group receiving a diet with 8% FRSM; FR/FS—group receiving a diet with 6% FRSM and 2% FSBM; FS/FR—group receiving a diet with 6% FSBM and 2% FRSM; FS—group receiving a diet with 8% FSBM.

The content of basic nutrients in the diets for weaners was similar in all experimental groups (Table 1) and was in line with the recommendations of the NRC^12^. The content of phytate phosphorus in the experimental diets with fermented components was lower than in the control. The reverse was noted for lactic acid^10^. The glucosinolate content was higher in the feeds with FRSM than in those with soybean meal (C) and FSBM (Table 1)^10^.

### Production parameters and digestibility of nutrients

The weaners from the control group and the group receiving 8% FSBM (group FS) had significantly lower body weight from 42 to 77 days of age than the weaners in group FR/FS (Table 2). The use of 6% FRSM and 2% FSBM in the diet had a positive effect on average daily gains. From 36 to 77 days, the highest daily gains were noted in group FR/FS (Table 2). Between 36 and 42 days, the piglets from group FR/FS had significantly higher daily feed intake than the animals from the other groups. In the following period, between days 43 and 49, the animals from group FR had significantly lower daily feed intake than the other groups. It should be noted that between 50 and 77 days of age, the feed intake was significantly lower in the FR, FR/FS, and FS groups than in the control group (Table 2). The piglets from the FR and FR/FS groups had the highest feed conversion rate (FCR) (Table 2).

**Table 2:** Body weight, ADG, DFI, FCR, mortality, and diarrhoea incidence in piglets.

	Feeding groups^1^					
	C	FR	FR/FS	FS/FR	FS	SEM
**Body weight (BW) (kg)**						
At 29 days	5.62	5.65	5.73	5.67	5.65	0.656
At 35 days	7.61	7.71	7.89	7.68	7.62	0.547
At 42 days	10.24^b^	10.69^a^	10.96^a^	10.51^ab^	10.27^b^	0.801
At 49 days	13.88^b^	14.43^ab^	14.89^a^	14.42^ab^	14.27^b^	0.765
At 77 days	33.01^b^	34.37^a^	34.72^a^	33.68^b^	33.79^b^	1.09
**Average daily gains (ADG) (kg)**						
29–35 days	0.284	0.294	0.308	0.287	0.281	0.033
36–42 days	0.376^b^	0.426^a^	0.438^a^	0.404^ab^	0.379^b^	0.076
43–49 days	0.520^b^	0.534^b^	0.561^a^	0.559^ab^	0.571^a^	0.087
50–77 days	0.683^b^	0.712^a^	0.708^a^	0.688^ab^	0.697^ab^	0.056
29–77 days	0.559^b^	0.586^a^	0.592^a^	0.572^ab^	0.574^ab^	0.077
**Daily feed intake (DFI) (kg)**						
29–35 days	0.321	0.327	0.338	0.329	0.337	0.054
36–42 days	0.561^b^	0.554^b^	0.632^a^	0.537^b^	0.573^b^	0.098
43–49 days	0.809^a^	0.724^c^	0.759^b^	0.764^b^	0.764^b^	0.111
50–77 days	1.139^a^	1.115^b^	1.116^b^	1.126^ab^	1.111^b^	0.087
29–77 days	0.892^a^	0.866^b^	0.885^a^	0.876^ab^	0.874^ab^	0.081
**Feed conversion ratio (FCR) (kg/kg)**						
29–35 days	1.13	1.11	1.10	1.15	1.20	0.074
36–42 days	1.49^a^	1.30^b^	1.44^ab^	1.33^b^	1.51^a^	0.121
43–49 days	1.56^a^	1.36^b^	1.35^b^	1.37^b^	1.34^b^	0.099
50–77 days	1.67^a^	1.57^b^	1.58^b^	1.64^ab^	1.59^ab^	0.087
29–77 days	1.60^a^	1.48^b^	1.50^b^	1.53^ab^	1.52^ab^	0.100
Mortality up to 77 days of age (%)	8.23^a^	4.31^b^	4.23^b^	4.89^b^	5.23^b^	0.47
Diarrhoea incidence (%)	17.42^a^	9.18^b^	8.58^b^	10.27^b^	11.42^b^	1.45
Days with diarrhoea	5.9^a^	3.2^b^	3.5^b^	4.1^ab^	4.4^ab^	0.28
Faeces consistency, points	2.4	1.8	1.9	2.1	2.2	0.23

^a,b,c^Values in rows marked with different letters differ significantly at p ≤ 0.05.

^1^Feeding groups: C—control; FR—group receiving a diet with 8% FRSM; FR/FS—group receiving a diet with 6% FRSM and 2% FSBM; FS/FR—group receiving a diet with 6% FSBM and 2% FRSM; FS—group receiving a diet with 8% FSBM.

Group C piglets had significantly higher mortality up to 77 days of age and a higher incidence of diarrhoea, compared to the experimental groups (Table 2). The piglets from groups FR and FR/FS had a significantly lower incidence of diarrhoea and fewer days with diarrhoea than the animals from the control group (Table 2).

The piglets from groups FR and FR/FS had significantly higher coefficients of apparent total tract digestibility (ATTD) and apparent ileum digestibility (AID) for crude protein (approx. 2.5 and 3.3%), methionine (6.4 and 4.6%), and ether extract (approx. 5.1 and 2.8%), compared to the control group (C). The piglets from groups FR, FR/FS, and FS had significantly higher coefficients of ATTD for crude fibre, compared to the control group (C). No such relationship was found in the case of crude fibre AID (Table 3). The addition of fermented soybean meal (FS/FR, FS) increased the ATTD and AID of lysine and threonine but only the ATTD of tryptophan, compared to the control group (C). The inclusion of the fermented feed in the diet significantly increased the ATTD and AID of calcium and phosphorus, with FRSM having a substantially greater effect than FSBM (Table 3).

**Table 3:** Apparent total tract digestibility (ATTD) and apparent ileum digestibility (AID) coefficients (%) of nutrients and selected minerals in piglets.

Item	Feeding groups^1^					
	C	FR	FR/FS	FS/FR	FS	SEM
**ATTD**						
Ether extract	60.04^b^	63.26^a^	62.92^a^	61.29^ab^	63.54^a^	1.02
Crude fibre	13.49^b^	15.28^a^	15.63^a^	14.27^ab^	15.39^a^	0.74
Nitrogen free extract	89.55	90.46	90.14	89.86	90.59	0.57
Crude protein	78.87^b^	80.98^a^	80.33^a^	79.19^ab^	79.78^ab^	0.82
Lys	82.88^b^	84.03^ab^	84.28^ab^	85.24^a^	85.41^a^	0.75
Met	79.87^c^	85.20^a^	84.77^a^	83.43^ab^	82.98^b^	0.64
Thr	78.97^b^	80.75^ab^	80.27^ab^	80.84^a^	81.09^a^	0.73
Trp	78.98^b^	80.93^ab^	81.12^ab^	81.98^a^	82.18^a^	0.49
Phosphorus	35.65^d^	51.22^a^	50.38^a^	47.55^b^	44.87^c^	0.65
Calcium	48.19^c^	56.55^a^	55.53^a^	52.98^b^	52.37^b^	0.81
**ADI**						
Ether extract	57.85^b^	59.32^a^	59.63^a^	58.44^ab^	59.32^a^	0.94
Crude fibre	4.59	5.12	5.13	4.95	5.16	0.17
Nitrogen free extract	86.12	86.84	86.25	87.11	87.17	0.21
Crude protein	73.84^b^	76.41^a^	76.14^a^	76.23^a^	75.31^ab^	0.24
Lys	80.14^b^	81.74^ab^	82.68^a^	82.71^a^	82.83^a^	0.29
Met	79.03^b^	82.52^a^	82.71^a^	81.11^ab^	81.39^ab^	0.31
Thr	75.24^b^	77.87^a^	76.42^ab^	77.96^a^	78.12^a^	0.23
Trp	77.29	77.69	78.11	78.24	78.42	0.28
Phosphorus	29.46^b^	42.88^a^	42.72^a^	42.17^a^	42.18^a^	0.21
Calcium	40.95^b^	46.51^a^	46.56^a^	45.98^a^	46.13^a^	0.38

^a,b,c,d^Values in rows marked with different letters differ significantly at p ≤ 0.05.

^1^Feeding groups: C—control; FR- group receiving a diet with 8% FRSM; FR/FS—group receiving a diet with 6% FRSM and 2% FSBM; FS/FR—group receiving a diet with 6% FSBM and 2% FRSM; FS—group receiving a diet with 8% FSBM.

### Results of the microbiological and histological analysis of the gastrointestinal tract

The animals from the FR/FS group had a significantly higher total bacterial count in the contents of the ileum. The bacterial count in the faeces was significantly higher in the control group than in the other groups (Table 4).

**Table 4:** Microbial composition (CFU/g) of the ileum and faeces of piglets.

Item	Feeding groups^1^					
	C	FR	FR/FS	FS/FR	FS	SEM
**Ileum**						
Total number of bacteria	1.3 × 10^5b^	1.4 × 10^5b^	6.8 × 10^6a^	2.8 × 10^5b^	1.4 × 10^4d^	5.4 × 10^5^
Total number of fungi	3.7 × 10^3a^	1.3 × 10^3c^	3.5 × 10^3ab^	3.6 × 10^3ab^	3.2 × 10^3b^	2.4 × 10^3^
Total number of coliforms	2.3 × 10^5a^	1.2 × 10^5b^	1.5 × 10^4d^	4.0 × 10^4c^	2.1 × 10^5a^	2.3 × 10^4^
Total number of *Escherichia* coli	3.2 × 10^5a^	1.2 × 10^5c^	1.3 × 10^4e^	5.1 × 10^4d^	2.5 × 10^5b^	3.1 × 10^4^
**Faeces**						
Total number of bacteria	3.4 × 10^5a^	3.7 × 10^4c^	8.5 × 10^4b^	2.7 × 10^4c^	8.1 × 10^3d^	3.3 × 10^4^
Total number of fungi	7.2 × 10^2a^	5 × 10^1c^	4.1 × 10^1 cd^	1.0 × 10^2b^	1.4 × 10^1d^	7.3 × 10^1^
Total number of coliforms	1.4 × 10^5a^	2.5 × 10^4b^	2.6 × 10^4b^	1.5 × 10^4c^	7.3 × 10^3d^	1.3 × 10^4^
Total number of *Escherichia* coli	8.7 × 10^4a^	2.6 × 10^4b^	2.4 × 10^4b^	1.5 × 10^4c^	7.3 × 10^3d^	7.7 × 10^3^
Total number of anaerobic bacteria *Clostridium perfringens*	1.7 × 10^6a^	1.5 × 10^6b^	2.0 × 10^5d^	6.6 × 10^5c^	1.2 × 10^6b^	1.5 × 10^5^

^a,b,c,d^Values in rows marked with different letters differ significantly at p ≤ 0.05.

^1^Feeding groups: C—control; FR—group receiving a diet with 8% FRSM; FR/FS—group receiving a diet with 6% FRSM and 2% FSBM; FS/FR—group receiving a diet with 6% FSBM and 2% FRSM; FS—group receiving a diet with 8% FSBM.

The total fungal count in the ileum contents was significantly higher in the control group animals vs. animals from the FS and FR groups, between which there was also a statistically significant difference (FS > FR). In the faeces of the piglets from the control group, the number of fungi was significantly higher than in the other groups (Table 4).

The inclusion of the fermented component in the feed for the piglets affected the total number of coliforms and *Escherichia coli* bacteria. The total number of these bacteria in the ileum was significantly higher in the control group and, in the case of coliforms, also in the group with 8% FSBM supplementation (group FS), compared to the other groups (Table 4).

The number of coliforms and *E. coli* in the faeces, as in the ileum, was significantly higher in the control animals than in the piglets receiving diets with fermented components (C vs. FR, FR/FS, FS/FR, and FS). The lowest numbers of both coliforms and *E. coli* were recorded in group FS (FS < FS/FR < FR = FR/FS). Significant differences were also noted in the number of anaerobic bacteria *Clostridium perfringens* in the faeces, which was significantly higher in group C than in the other groups, with significant differences between them: FR/FS < FS/FR < FR = FS (Table 4).

The fermented component used in the feed in the amounts of 6% FRSM and 2% FSBM increased the length and width of the villi in the ileum, compared to the other groups (Table 5). The shortest villi were noted in groups C and FS/FR, and the narrowest villi were observed in group C. The deepest crypts in the ileum were found in groups C and FR vs. groups FR/FS and FS/FR. The group C animals had the widest crypts, compared to the other groups, between which there were statistically significant differences, i.e. FR = FR/FS < FS/FR = FS. It should also be noted that the width of the mucosa was significantly greater in the FR/FS group than in group C (Table 5). The inclusion of FRSM in the feed significantly reduced the depth and width of the crypts in the caecum and colon compared to the control group. A reverse relationship was observed for the width of the caecum mucosa (Table 5). The width of the colonic mucosa was also significantly smaller in the control group piglets (C) than in the animals from the groups receiving feed supplemented with the fermented components (Table 5).

**Table 5:** Histological appearance and intestinal morphology in piglets (µm).

Item	Feeding groups^1^					
	C	FR	FR/FS	FS/FR	FS	SEM
**Ileum**						
Villi						
Length	408.0^c^	436.9^b^	508.4^a^	397.6^c^	444.9^b^	3.32
Width	146.2^c^	155.2^b^	163.9^a^	153.9^b^	155.9^b^	2.18
Crypts						
Depth	245.4^a^	264.6^a^	209.9^b^	206.2^b^	232.8^ab^	3.06
Width	56.19^a^	43.16^c^	44.77^c^	49.87^b^	47.13^bc^	1.03
Mucosa						
Thickness	28.33^b^	30.71^ab^	32.69^a^	30.99^ab^	29.14^ab^	1.11
**Caecum**						
Crypts						
Depth	438.5^a^	410.5^b^	391.6^b^	380.7^b^	425.34^ab^	2.02
Width	76.98^a^	67.21^b^	65.98^b^	66.98^b^	70.54^ab^	1.93
Mucosa						
Thickness	233.4^b^	265.9^a^	270.9^a^	266.1^a^	250.7^ab^	4.98
**Colon**						
Crypts						
Depth	468.5^a^	409.1^b^	426.7^b^	425.1^b^	436.1^ab^	2.34
Width	64.98^a^	57.98^b^	58.99^b^	59.63^b^	60.63^ab^	1.97
Mucosa						
Thickness	189.3^c^	226.9^a^	222.9^a^	200.7^b^	231.2^a^	3.94

^a,b,c^Values in rows marked with different letters differ significantly at p ≤ 0.05.

^1^Feeding groups: C—control; FR—group receiving a diet with 8% FRSM; FR/FS—group receiving a diet with 6% FRSM and 2% FSBM; FS/FR—group receiving a diet with 6% FSBM and 2% FRSM; FS—group receiving a diet with 8% FSBM.

## Discussion

Despite their high nutrient content and beneficial amino acid composition, soybeans are not entirely safe feed components for piglets. Their presence in feed introduces a number of anti-nutritional substances, such as protease inhibitors, β-conglycinins, and phytates, which significantly limit feed efficiency in monogastric animals. Fermentation of soybeans, which reduces the content of anti-nutritional substances, can be a solution to this problem^13,14^. Fermentation reduces the content of glycine and β-conglycinin, which are potential antigenic and allergenic compounds and can cause villous atrophy and crypt hyperplasia in the ileum of weaned piglets. During fermentation of soybean meal, protein and carbohydrates are partially degraded to low-molecular-weight compounds, thereby increasing their solubility in water, which facilitates digestion and improves production results^15^. In our experiment, no satisfactory results were obtained using 8% FSBM relative to the control group with toasted SBM. Only the digestibility coefficients of basic nutrients in the group of piglets receiving diets with FSBM were higher, while body weight and FCR were comparable to the control group. Piglets receiving 8% FSBM in their feed had lower daily feed intake than the group C animals, possibly due to the presence of lactic acid, which can adversely affect its palatability^5^, leading to a decrease in average daily intake. However, research conducted by some authors^16^ indicates that FSBM products improve the digestibility of basic nutrients, which corresponds to improvement in piglet production parameters. Jones et al*.*^13^ reported an increase in the digestibility of His, Lys, and Thr and an increase in average daily gains (ADG) in piglets receiving diets with various FSBM levels (1.75%, 3%, or 6%), compared to piglets receiving blood plasma. Slightly different results were obtained by Cho et al.^16^ and Kim et al*.*^14^, who used various levels of FSBM in piglet feed and noted an increase in the ratio of daily weight gain to daily feed intake (G:F). Cho et al.^16^ showed that although a diet supplemented with FSBM improved His and Lys digestibility and the G:F ratio, it did not improve ADG, which is consistent with our observations. Further research is required to draw definitive conclusions on the effect of FSBM on the efficiency of piglet rearing. The comparable performance effects in groups FS vs. C may indicate that toasting and fermentation similarly reduce the content of anti-nutrients found in soybean meal, which may suggest that the combined use of these two treatments is ungrounded.

The coefficients for ATTD calculated in the present study were significantly higher, especially for crude fibre, than the AID values. This is linked to microbiological decomposition in the large intestine and the resorption of generated metabolites, as confirmed by other authors^3,7,14^.

Fermentation is also an effective method of increasing the use of cheaper rapeseed meal, which may reduce pressure on other more expensive and controversial protein feed resources (GMO soybean meal). The rapeseed meal obtained in this matter also supplies valuable biologically active substances, including proteolytic enzymes, bactericides, and antioxidant peptides^6^. Low-molecular-weight peptides, referred to as antioxidant peptides, formed during fermentation of rapeseed meal, exhibit high free-radical-scavenging activity and inhibit lipid peroxidation, while at the same time helping to preserve the structural integrity of the cell membrane^17^. This may improve nutrient absorption from the intestinal contents, and may also have a beneficial effect on the development of the intestinal microbiota^18^. Substantial improvement in production parameters was recorded in piglets receiving the FRSM-supplemented diet (group FR) compared to the control group animals. The significant increase in the ATTD and AID coefficients of protein, fat, and amino acids, compared to the animals from the control group, corresponded to an increase in final body weight, lower feed intake and, most important, a higher FCR in this group of animals (FR). As suggested by Xu et al.^15^, this may be associated with the effect of fermentation improving the availability of essential amino acids (Lys, Met + Cys) from rapeseed meal, a reduction in the content of anti-nutrients, and enhanced absorption of short peptides.

The beneficial effect of FRSM can also be attributed to a decrease in the amount of non-starch polysaccharides (NSP), lignin, and phytates, leading to an increase in carbohydrate digestibility^19^ and availability of minerals, including phosphorus and calcium^20^.

Fermentation of protein feed components lowers their pH, increases the amount of lactic acid bacteria and the lactic acid they produce (which was seen in our experiment), and leads to the production of certain enzymes and vitamins^21^. The inclusion of FRSM and/or FSBM in the diet for piglets enriches the gut microbiota with a greater amount of lactic acid. In line with our observations, Canibe and Jensen^21^ and van Winsen et al*.*^22^ reported that supplementation with fermented feed increased the population of *Lactobacillus* bacteria in the stomach and intestines. A balanced population of microorganisms supports intestinal function, which helps to control pathogens in the digestive tract. This resulted in a significant reduction in the total number of coliforms and *E. coli* in the ileum and the total number of anaerobic *C. perfringens* in the faeces relative to the control group. This is confirmed by Grela et al*.*^23^, who found that a decrease in feed pH to 4.0–4.5 stimulated the development of beneficial gastrointestinal microorganisms simultaneously reducing pathogenic microbes. As shown by Kiers et al*.*^24^, fermented components are a source of certain microorganisms that can inhibit colonization of the intestine by pathogens responsible for diarrhoea in pigs. The significant reduction in the number of pathogenic bacteria (*Enterobacteriaceae*) in the caecum, colon, and rectum of pigs receiving fermented feed is linked not only to the decrease in pH but to other factors as well, such as higher availability of nutrients, competition for receptor sites, and immune response.

Limitation of the activity of pathogenic *E. coli* strains and the occurrence of diarrhoea improves utilization of feed nutrients and production results, especially in weaned piglets^25^. A reduction in pathogenic bacteria was observed in all animals receiving diets with fermented components. As suggested by Kiers et al.^24^, the negative effects can be minimized by using feeds with fermented components.

The improvement in production results in pigs receiving fermented (dry or wet) feed is closely linked to modification of the intestinal epithelium and an increase in its absorption surface, mainly through an increase in the length of the intestinal villi^23,25,26^. Intestinal villi are the main absorption site, and their elongation is beneficial for nutrient resorption^26–28^. For this reason, intestinal morphology, i.e. villus height, crypt depth, ratio of villus depth to crypt depth, and intestinal wall thickness, are valuable indicators of the absorption capacity of the ileum. Another indicator of the body’s level of nutrition and the availability of energy is the thickness of the muscularis mucosae. Improvement in these parameters was achieved in our experiment in piglets receiving a diet with 6% FRSM and 2% FSBM, which had a beneficial effect on production parameters and the ATTD and AID coefficients of nutrients, especially protein and fat. This may indicate mutual additivity of beneficial nutrients from soybean and rapeseed arising as a result of fermentation. Moreover, the improvement observed in the intestinal morphology may be primarily due to the removal of anti-nutritional substances and degradation of large proteins to low-molecular-weight peptides in fermented rapeseed oil^17^.

Digestion and absorption are believed to improve as villus height increases relative to crypt depth^28^. Improvement of the villi/crypt ratio in pigs receiving fermented feed has also been reported by other authors^26^. The improvement in intestinal morphology may have been associated with the presence of lactic acid in fermented feed, which contributes to the growth of villi, improves their shape, and increases the ratio of villus height to crypt depth, as shown by Scholten et al*.*^26^.

Research conducted by Hong et al*.*^29^ confirmed the hypothesis that piglets fed liquid fermented feed had the highest content of acetic and lactic acid in the stomach, ileum, and colon. Scholten et al.^26^ suggest a possible relationship between the concentration of organic acids in the intestinal lumen, the source of which is fermented components, and an increase in the size of villi.

## Conclusions

The results of the study confirm the hypothesis that feeding piglets a diet supplemented with fermented components, in particular with 8% FRSM or 6% FRSM and 2% FSBM, has a positive effect on the microbiological composition and histology of the intestines, which results in improved nutrient digestibility coefficients and better production results. The use of dried fermented protein components in the feed ration for piglets improves the functioning of the body, especially the gut microbiota, and thus positively affects their body condition, which is highly beneficial in pig rearing.

## Methods

The study was carried out in compliance with the ARRIVE guidelines.

All methods were carried out in accordance with relevant guidelines and regulations regarding to life animal studies and the procedures were complied with the Directive 2007/526/EC of the European Parliament and of the Council on the protection of animals used for scientific purposes.

The experimental procedures used throughout this study were approved by the II Local Ethics Committee on Animal Experimentation of University of Life Sciences in Lublin, Poland (Resolution No. 21/2016).

### Animals and experimental design

The study was conducted on 150 28-day-old weaned piglets divided into 5 analogous groups in terms of body weight and sex. Each group comprised 30 weaned piglets (15 gilts and 15 barrows), which were placed in 15 pens with 2 piglets in each (1 gilt and 1 barrow). The piglets were housed in a building with controlled environmental conditions and received crumble feed in an identical feeding system to approximately 30 kg body weight. From 29 to 42 days of age, the piglets were fed prestarter mixtures ad libitum. From 43 days of age, the animals were fed starter diets ad libitum and kept in standard rearing conditions with constant access to fresh water.

Piglets in the control group (C) received standard complete prestarter and starter diets with soybean meal (SBM). Animals in group FR received feed in which a portion of SBM was replaced with 8% fermented dried rapeseed meal (FRSM). Animals in group FR/FS received a diet in which some of the SBM was replaced with 6% FRSM and 2% fermented dried soybean meal (FSBM). Piglets in group FS/FR received a diet with 2% FRSM and 6% FSBM. Animals in group FS received a diet with 8% FSBM. The fermentation process used 90% rapeseed meal or soybean meal and 10% carbohydrates additives (7% steamed potatoes and 3% wheat). *Bacillus subtilis* and *Lactobacillus fermentum*, were used as main fermentation strains. The exact course of the fermentation process is protected by a patent. The ingredient composition of the complete diets is presented in Table 6^10^.

**Table 6:** Ingredient composition (% of air-dry matter) of piglet diets^10^.

Diets	Prestarter (29–42 days)					Starter (43–77 days)				
Groups	C	FR	FR/FS	FS/FR	FS	C	FR	FR/FS	FS/FR	FS
Wheat	35.5	35.5	35.5	35.5	35.5	40.0	40.0	40.0	40.0	40.0
Barley	28.0	26.0	27.0	29.0	30.0	28.0	26.0	27.0	29.0	30.0
Soybean meal, 44% CP	16.0	10.0	9.0	7.0	6.0	16.0	10.0	9.0	7.0	6.0
Dried whey, 16% CP	4.0	4.0	4.0	4.0	4.0	0.0	0.0	0.0	0.0	0.0
Soybean oil	2.5	2.5	2.5	2.5	2.5	2.5	2.5	2.5	2.5	2.5
Complementary feed^a–e^	8.5	8.5	8.5	8.5	8.5	8.0	8.0	8.0	8.0	8.0
Mineral-vitamin premix^f^	5.0	5.0	5.0	5.0	5.0	5.0	5.0	5.0	5.0	5.0
Acidifier^g^	0.5	0.5	0.5	0.5	0.5	0.5	0.5	0.5	0.5	0.5
FRSM	0.0	8.0	6.0	2.0	0.0	0.0	8.0	6.0	2.0	0.0
FSBM	0.0	0.0	2.0	6.0	8.0	0.0	0.0	2.0	6.0	8.0

*C* control, *FR* group receiving a diet with 8% FRSM, *FR/FS* group receiving a diet with 6% FRSM and 2% FSBM, *FS/FR* group receiving a diet with 6% FSBM and 2% FRSM, *FS* group receiving a diet with 8% FSBM.

^a^Complementary feed, 1 kg (control group) containing crude protein 36.15%, lysine 2.50%, methionine 0.73%, crude fat 14.3%, crude fibre 2.85%, calcium 1.2%, phosphorus 0.85%, sodium 0.35%, BHT 280 mg.

^b^Complementary feed, 1 kg (FR group) containing crude protein 36.65%, lysine 2.61%, methionine 0.75%, crude fat 14.6%, crude fibre 2.75%, calcium 1.15%, phosphorus 0.84%, sodium 0.33%, BHT 280 mg.

^c^Complementary feed, 1 kg (FR/FS group) containing crude protein 36.60%, lysine 2.58%, methionine 0.76%, crude fat 14.5%, crude fibre 2.77%, calcium 1.17%, phosphorus 0.84%, sodium 0.33%, BHT 280 mg.

^d^Complementary feed, 1 kg (FS/FR group) containing crude protein 36.25%, lysine 2.50%, methionine 0.75%, crude fat 14.3%, crude fibre 2.85%, calcium 1.2%, phosphorus 0.84%, sodium 0.35%, BHT 280 mg.

^e^Complementary feed, 1 kg (FS group) containing crude protein 36.05%, lysine 2.49%, methionine 0.74%, crude fat 14.3%, crude fibre 2.90%, calcium 1.22%, phosphorus 0.85%, sodium 0.36%, BHT 280 mg.

^f^1 kg mineral-vitamin premix containing: calcium 130 g, phosphorus 50 g, sodium 35 g, magnesium 4.0 g, lysine 89 g, methionine 46 g, vitamin A 300,000 IU, vitamin D_3_ 40,000 IU, vitamin E 2600 mg, calcium iodide 32 mg, selenium 8 mg, copper 3.2 g, iron 2.4 g, zinc 2.4 g, manganese 2976 mg, 25,000 FTU.

^**g**^1 kg acidifier containing orthophosphoric acid 320 g, citric acid 110 g, fumaric acid 50 g, propionic acid 45 g, formic acid 45 g, carrier (silicon dioxide) 430 g.

The effectiveness of the diet was assessed by monitoring piglets’ weight, daily feed intake, and body condition. The piglets were weighed at 29, 36, 43, 50, and 77 days of age. The portions of feed were weighed out and placed in automatic feeders, and any uneaten feed was recorded on a daily basis. The animals’ body condition was observed throughout the experiment.

Diarrhoea symptoms and mortality, if any, were recorded twice a day during rearing for each pierced piglet. Faeces were assessed visually and assigned a score according to the following scale: 1—firm and well-formed faeces, 2—loose and shapeless faeces, 3—runny faeces, and 4—watery diarrhoea^30^. The percentage of diarrhoea cases in relation to the total number of piglets in the group (diarrhoea incidence) and the average duration of diarrhoea in days were calculated.

Apparent total tract digestibility (ATTD) of nutrients was measured in six barrows per group in metabolic cages in the period from 64 to 70 days of age (average body weight of piglets per group). At the end of the trial, the six piglets were slaughtered and ileal digesta was collected for determination of digestibility (AID).

The slaughter was conducted in accordance with the technology currently employed in the meat industry. Following electrical stunning, the pigs were killed by bleeding, by severing the blood vessels of the throat.

The contents of the ileum were collected for microbiological tests, and tissue samples from the gastrointestinal tract (ileum, caecum and colon) were collected for histological examination. The samples were fixed in 10% buffered formalin and stored at approx. − 4 °C.

### Determination of nutrient digestibility

Digestibility (ATTD) of nutrients was determined for six consecutive days using an endogenous indicator (4 mol/L HCl-insoluble ash) as an indigestible marker.

About 0.3 kg of faeces was collected in the morning from six animals in each group. The faeces were weighed, and samples from 6 days were collected in a covered container with a few drops of sulphuric acid and frozen. Next, the faeces were mixed and 10 g of each sample was taken for determination of total protein content^30,31^. The remainder of the faeces were oven-dried (60 °C/72 h) to constant weight.

After slaughter, the abdominal cavity was opened, and a 0.5‒0.6 m piece of the ileum, backwards from the ileocaecal junction, was isolated. Digesta was collected from the isolated intestine and, if that part of the ileum was empty, an additional 0.5‒0.6 m piece was isolated and the digesta was collected. The digesta was frozen immediately. Digesta samples were freeze-dried. From each such portion of dried faeces and freeze-dried ileum samples, finely ground in a mill, two samples were weighed out for analysis of dry matter, crude ash, crude protein, crude fat, and crude fibre, amino acids, minerals (P and Ca), and markers (4 mol/L HCl-insoluble ash).

The concentrations of nutrients and 4 mol/L HCl-insoluble ash were determined^30,31^.

The calculation was made as follows:

$$ \begin{aligned}  {\text{AID}}\;{\text{or}}\;{\text{ATTD}} & = 100 - 100 \times (({\text{indicator}}\;{\text{content}}\;{\text{in}}\;{\text{diet}} \\   & \quad \times {\text{nutrient}}\;{\text{content}}\;{\text{in}}\;{\text{faeces}}\;{\text{or}}\;{\text{ileum}}\;{\text{samples}})/({\text{indicator}} \\   & \quad \quad {\text{content}}\;{\text{in}}\;{\text{faeces}}\;{\text{or}}\;{\text{ileum}}\;{\text{samples}} \times {\text{nutrient}}\;{\text{content}} \\   & \quad \quad {\text{in}}\;{\text{diet}})). \\ \end{aligned} $$

### Laboratory analyses

Dry matter, crude ash, crude protein, crude fat, and crude fibre were determined in the diets, fermented soybean and rapeseed meal, faeces, and ileum samples^10,30,31^. Amino acids were analysed with a Sykam Amino Acid Analyzer (Laserchrom HPLC Laboratories Ltd. Inc., Rochester, UK) following hydrolysis of the samples with 6 N HCl for 24 h at 110 °C. Methionine was analysed as Met sulphone, with hydrolysis preceded by cold performic acid oxidation overnight. Tryptophan was determined following NaOH hydrolysis for 22 h at 110 °C according to the AOAC procedure^31^. The content of calcium, sodium, iron, copper, and zinc was determined by atomic absorption spectrometry with a Varian model 720-ES ICP-OES spectrophotometer (Varian, Palo Alto, USA), Total P content in the feed, faeces, and ileum samples was determined colorimetrically with a Helios Alpha UV–Vis spectrophotometer (Spectronic Unicam, Leeds, UK). The contents of phytate phosphorus, lactic acid, glucosinolates, and tannins were determined in the diets.

Phytate was determined colorimetrically based on the pink colour of the Wade reagent, which is formed upon the reaction of ferric ions and sulfosalicylic acid and has an absorbance maximum at 500 nm. In the presence of phytate, iron is sequestered and is unavailable to react with sulfosalicylic acid, resulting in a decrease in the intensity of the pink colour^10,32^.

The content of lactic acid was determined colorimetrically by the LA-Fe (III) complex method. The lactic acid-iron (III) complex was formed as a result of the acid-iron (III) chloride reaction, and its absorbance was measured at 410 nm using a Helios Alpha UV–Vis spectrophotometer (Spectronic Unicam, Leeds, UK)^10,33^.

The glucosinolate content in the samples was estimated according to the standard^10,34^ using high performance liquid chromatography using the Agilent 1100 Series HPLC system, on an ODS Agilent Zorbax column (5 µl 4.6 × 250 mm), with a UV–Vis detector, 229 nm. An injection volume of 50 µl (Agilent Autosampler) and reversed phase gradient elution A: H_2_O, B: Acetonitrile: H_2_O (20:80 v/v) were used. The flow rate was 1.0 ml/min and the analysis time was 43 min.

The Folin-Denis spectrophotometric method was used to determine the tannin content according to Canbaş et al*.*^35^ with modification^10^. A measured weight of each sample (0.5 g) was dispersed in 35 ml of distilled mixtures (ethanol:glycerin:water in a 1:1:1 ratio) for 30 min at 60 °C, with shaking every 5 min. After the 30 min, it was centrifuged and the extract was obtained. A 1 ml volume of the supernatant (extracts) was transferred into a 50 ml volumetric flask. Similarly, 1 ml of a standard tannic acid solution was transferred into a separate 50 ml flask. A 2.0 ml volume of the Folin-Denis reagent was added to each flask, followed by 10 ml of a saturated Na_2_CO_3_ solution. The mixture was diluted to the mark in the flask (50 ml) and incubated for 15 min at room temperature. The absorbances were measured at 700 nm in a Helios Alpha UV–Vis spectrophotometer (Spectronic Unicam, Leeds, UK). Readings were taken with the reagent blank at zero.

### Microbiological analysis of gastrointestinal contents

During slaughter, the contents of the final segment of the ileum of six piglets per group were collected for microbiological testing. The material was cooled to 6–8 °C and transported to the laboratory (about 1 h). Then, one gram was weighed out from each sample and inoculated into 9 ml of Ringer's solution with Tween 80 and homogenized. Decimal dilutions of each sample were made according to ISO 6887-1^36^. Each dilution was plated on previously prepared sterile solid media in the amount of 100 μl and then incubated according to the standards^30^.

The following parameters were determined in the material:

- total bacterial count—the material was plated on tryptic soy agar (TSA) and incubated for 72 (± 3) h in a thermostat (30 ± 2 °C) according to PN-EN ISO 4833-2^37^.
- the total number of yeasts was determined on an agar medium with dichlorane and 18% glycerol (DG 18) incubated for 5–7 days in a thermostat (25 ± 1 °C) according to PN-ISO 21527-1/2^38^.
- total number of coliform bacteria—the material was plated on Violet Red Bile Lactose (VRBL) agar and incubated for 24 ± 2 h in a thermostat (37 ± 2 °C) according to PN-ISO 4832:2007^39^.
- total number of *E. coli* bacteria—the material was plated on Tryptone Bile X-Glucuronide Medium (TBX) and then incubated for 24 h in a thermostat (44 ± 3 °C) according to PN-ISO-16649–2^40^.
- total number of *C. perfringens* bacteria—the material was plated on Tryptose Sulphite Cycloserine Agar and incubated for 20 ± 2 h at 37 °C (± 1 °C) in anaerobic conditions obtained using GasPak Plus (Anaerobic System Envelopes with Palladium Catalyst, BD BBL), which is a system for producing gas environments, according to PN-EN ISO 7937^41^. Each culture on solid substrates was conducted in duplicate. The numbers of microorganisms were expressed as colony forming units (CFU) per gram of the tested material. The result for one animal was expressed as the mean of replicates of the CFU count per g of faeces.

### Histomorphometry

Tissue samples from the ileum, caecum, and colon were embedded in paraffin, and serial histological sections (5 μm thick) were stained with haematoxylin and eosin for histomorphometric analysis under a light microscope. For each piglet (6 barrows per group), villus height, crypt depth, and the thickness of the tunica mucosa and tunica muscularis were measured in 5–8 slides for each section of the intestine (ileum, caecum, and colon) with an optical microscope (× 4 or × 10 objectives, Olympus BX 61, Warsaw, Poland) coupled via a digital camera to a PC equipped with Cell P (Olympus) software. For each intestinal segment from each piglet, 30 well-oriented villi and crypts on the slide as well as the thickness of the tunica mucosa and tunica muscularis (30 measurement points each) were measured. The mean value of each of the parameters was used to represent the data for a single piglet. The procedure was repeated for each piglet, and statistical analysis was performed for the five groups of piglets (n = 6 in each experimental group)^30^.

### Statistical analysis

The numerical data were analysed by one-way analysis of variance (ANOVA), and the significance of differences between the groups was determined by the Tukey post-hoc test, assuming significance levels of 0.05 and 0.01. The pen was the experimental unit for feed intake and the feed conversion ratio (n = 15 per group). An individual piglet was the experimental unit for body weight and body condition (n = 30 per group). Six piglets were the experimental unit in the case of all other traits. The tables present the mean values and the standard error of the mean (SEM). The calculations were made using SAS 9.4 software (SAS Institute, Cary NC).

### Ethics statement

The experimental procedure was approved by the Local Ethics Commission for Experiments with Animals in Lublin, Poland (approval no. 21/2016).

## References

[1] De Visser CLM, Schreuder R, Stoddard F (2014). The EU's dependency on soya bean import for the animal feed industry and potential for EU produced alternatives. Oilseeds Fats Crops Lipids.

[2] Florou-Paneri P, Christaki E, Giannenas I, Bonos E, Skoufos I, Tsinas I (2014). Alternative protein sources to soybean meal in pig diets. J. Food Agric. Environ..

[3] Choct M, Dersjant-Li Y, McLeish J, Peisker M (2010). Soy oligosaccharides and soluble non-starch polysaccharides: A review of digestion, nutritive and anti-nutritive effects in pigs and poultry. Asian-Aust. J. Anim. Sci..

[4] Omosebi MO, Osundahunsi OF, Fagbemi TN (2018). Effect of extrusion on protein quality, antinutritional factors, and digestibility of complementary diet from quality protein maize and soybean protein concentrate. J. Food Biochem..

[5] Schőne F, Leiterer M, Hartung H, Jahreis G, Tischendorf F (2001). Rapeseed glucosinolates and iodine in sows affect the milk iodine concentration and the iodine status of piglets. Br. J. Nutr..

[6] Nkhata, SG, Ayua, E, Kamau, EH, Shingiro, JB. Fermentation and germination improve nutritional value of cereals and legumes through activation of endogenous enzymes. *Food Sci. Nutr.* **6**, 2446–2458, (2018). 10.1002/fsn3.846 PMC6261201 30510746

[7] Xu B, Li Z, Wang C, Fu J, Zhang Y, Wang Y (2020). Effects of fermented feed supplementation on pig growth performance: A meta-analysis. Anim. Feed Sci. Technol..

[8] Canibe N, Jensen BB (2012). Fermented liquid feed—Microbial and nutritional aspects and impact on enteric diseases in pigs. Anim. Feed Sci. Technol..

[9] Song YS, Pérez VG, Pettigrew JE, Martinez-Villaluenga C, de Mejia EG (2010). Fermentation of soybean meal and its inclusion in diets for newly weaned pigs reduced diarrhea and measures of immunoreactivity in the plasma. Anim. Feed Sci. Technol..

[10] Czech A, Sembratowicz I, Kiesz M (2021). The effects of a fermented rapeseed or/and soybean meal additive on antioxidant parameters in the blood and tissues of piglets. Animals.

[11] Kirchgessner M, Roth FX (1983). Schätzgleichungen zur Ermittlung des energetischen Futterwertes von Mischfuttermitteln für Schweine. J. Anim. Physiol. Anim. Nutr..

[12] National Research Council (NRC) (2012). Nutrient Requirements of Swine, 11th revised edition.

[13] Jones CK, DeRouchey JM, Nelssen JL, Tokach MD, Dritz SS, Goodband RD (2010). Effects of fermented soybean meal and specialty animal protein sources on nursery pig performance. J. Anim. Sci..

[14] Kim YG, Lohakare JD, Yun JH, Heo S, Chae BJ (2007). Effect of feeding levels of microbial fermented soy protein on the growth performance, nutrient digestibility and intenstinal morphology in weaned piglets. Asian-Aust. J. Anim. Sci..

[15] Xu FZ, Zeng XG, Ding XL (2012). Effects of replacing soybean meal with fermented rapeseed meal on performance, serum biochemical variables and intestinal morphology of broilers. Asian-Aust. J. Anim. Sci..

[16] Cho JH, Min BJ, Chen YJ, Yoo JS, Wang Q, Kim JD (2007). Evaluation of FSP (fermented soy protein) to replace soybean meal in weaned pigs: Growth performance, blood urea nitrogen and total protein concentrations in serum and nutrient digestibility. Asian-Aust. J. Anim. Sci..

[17] He R, Ju X, Yuan J, Wang L, Girgih AT, Aluko RE (2012). Antioxidant activities of rapeseed peptides produced by solid state fermentation. Food Res. Int..

[18] Shao BZ, Zhang W, Shi YX (2007). Downstream processes for aqueous enzymatic extraction of rapeseed oil and protein hydrolysates. J. Am. Oil Chem. Soc.

[19] Jakobsen GV, Jensen BB, Bach Knudsen KE, Brooks N (2015). Improving the nutritional value of rapeseed cake and wheatdried distillers grains with solubles by addition of enzymes during liquid fermentation. Anim. Feed Sci. Technol..

[20] Tomaszewska E, Muszyński S, Dobrowolski P, Kamiński D, Czech A, Grela ER (2019). Dried fermented post-extraction rapeseed meal given to sows as an alternative protein source for soybean meal during pregnancy improves bone development of their offspring. Livest. Sci..

[21] Canibe N, Jensen BB (2003). Fermented and non-fermented liquid feed to growing pigs: Effect on aspects of gastrointestinal ecology and growth performance. J. Anim. Sci..

[22] van Winsen RL, Urlings BAP, Lipman LJA, Snijders JMA, Keuzenkamp D, Verheijden JHM (2001). Effect of fermented feed on the microbial population of the gastrointestinal tracts of pigs. Appl. Environ. Microb..

[23] Grela ER, Czech A, Kusior G, Szczotka-Bochniarz A, Klebaniuk R (2018). The effect of feeding system and sex on the performance and selected gastrointestinal features of fattening pigs. Pol. J. Vet. Sci..

[24] Kiers JL, Meijer JC, Nout MJ, Rombouts FM, Nabuurs MJ, van der Meulen J (2003). Effect of fermented soya beans on diarrhoea and feed efficiency in weaned piglets. J. Appl. Microbiol..

[25] Pluske JR, Pethick DW, Hopwood DE, Hampson DJ (2002). Nutritional influences on some major enteric bacterial diseases in pigs. Nutr. Res. Rev..

[26] Scholten RH, van der Peet-Schwering CM, den Hartog LA, Balk M, Schrama JW, Verstegen MW (2002). Fermented wheat in liquid diets: Effects on gastrointestinal characteristics in weanling piglets. J. Anim. Sci..

[27] Thu TV, Loh TC, Foo HL, Yaakub H, Bejo MH (2011). Effects of liquid metabolite combinations produced by *Lactobacillus plantarum* on growth performance, feaces characteristics, intestinal morphology and diarrhea incidence in postweaning pigs. Trop. Anim. Health. Prod..

[28] Pluske JR, Thompson MJ, Atwood CS, Bird PH, Williams LH, Hartmenn PE (1996). Maintenance of villus height and crypt depth, and enhancement of disaccharide digestion and monosaccharide absorption, in piglets fed on cows’ whole milk after weaning. Brit. J. Nutr..

[29] Hong TTT, Thuy TT, Passoth V, Lindberg JE (2009). Gut ecology, feed digestion and performance in weaned piglets fed liquid diets. Livest. Sci..

[30] Pedersen KS, Toft N (2011). Intra- and inter-observer agreement when using a descriptive classification scale for clinical assessment of faecal consistency in growing pigs. Prev. Vet. Med..

[31] Grela ER, Czech A, Kiesz M, Wlazło Ł, Nowakowicz-Dębek B (2019). A fermented rapeseed meal additive: Effects on production performance, nutrient digestibility, colostrum immunoglobulin content and microbial flora in sows. Anim. Nutr..

[32] Association of Official Analytical Chemists (AOAC) (2012). Official Methods of Analysis of AOAC International.

[33] Vaintraub IA, Lapteva NA (1988). Colorimetric determination of phytate in unpurified extracts of seeds and the products of their processing. Anal Biochem.

[34] Taylor KACC (1996). A simple colorimetric assay for muramic acid and lactic acid. Appl. Biochem. Biotech..

[35] PN-ISO 10633-1 Oilseed residues - Determination of glucosinolates content - Method using high-performance liquid chromatography; Polish Committee for Standardization: Warsaw, Poland, (2000).

[36] Canbaş A, Erten H, Özşahin F (2001). The effects of storage temperature on the chemical composition of hop pellets. Process Biochem..

[37] ISO 6887-1 Microbiology of the food chain - Preparation of test samples, initial suspension and decimal dilutions for microbiological examination - Part 1: general rules for the preparation of the initial suspension and decimal dilutions. Ed. 2, pp 26, Technical Committee (2017).

[38] PN-ISO 4833-2:2013-12. Microbiology of the food chain. Horizontal method for the enumeration of microorganisms. Part 2: Colony-Count at 30 Degrees C by the surface plating technique; Polish Committee for Standardization: Warsaw, Poland, (2013).

[39] PN-ISO 21527-1:2009. Microbiology of food and animal feeding stuffs. Horizontal method for the enumeration of yeasts and moulds. Part 1: Colony-count technique in products with wather activity greater than 0.95; Polish Committee for Standardization: Warsaw, Poland, (2009).

[40] PN-ISO 4832 Microbiology of food and animal feeding stuffs e horizontal method for the enumeration of coliforms - colony-count technique. Polish Committee for Standardization: Warsaw, Poland, (2007).

[41] PN-ISO 16649-2:2004. Microbiology of food and animal feeding stuffs. Horizontal method for the enumeration of-glucuronidase-positive *Escherichia coli.* Part 2: Colony-count technique at 44 C using 5-bromo-4-chloro-3-indolyl-D-glucuronide; Polish Committee for Standardization: Warsaw, Poland, (2004).

[42] PN-EN ISO 7937 Microbiology of food and animal feeding stuffs - horizontal method for the enumeration of Clostridium perfringens e colony-count technique, Polish Committee for Standardization: Warsaw, Poland, (2005).

